# Isolation Syndrome after Cardiac Arrest and Therapeutic Hypothermia

**DOI:** 10.3389/fnins.2016.00259

**Published:** 2016-06-09

**Authors:** Peter B. Forgacs, Esteban A. Fridman, Andrew M. Goldfine, Nicholas D. Schiff

**Affiliations:** ^1^Feil Family Brain and Mind Research Institute, Weill Cornell Medical CollegeNew York, NY, USA; ^2^Department of Neurology, Weill Cornell Medical CollegeNew York, NY, USA; ^3^The Rockefeller UniversityNew York, NY, USA; ^4^Department of Neurology, SUNY Stony Brook Medical CenterStony Brook, NY, USA

**Keywords:** cognitive motor dissociation, disorders of consciousness, cardiac arrest, hypoxic brain injury, therapeutic hypothermia, quantitative electroencephalography, positron emission tomography

## Abstract

Here, we present the first description of an isolation syndrome in a patient who suffered prolonged cardiac arrest and underwent a standard therapeutic hypothermia protocol. Two years after the arrest, the patient demonstrated no motor responses to commands, communication capabilities, or visual tracking at the bedside. However, resting neuronal metabolism and electrical activity across the entire anterior forebrain was found to be normal despite severe structural injuries to primary motor, parietal, and occipital cortices. In addition, using quantitative electroencephalography, the patient showed evidence for willful modulation of brain activity in response to auditory commands revealing covert conscious awareness. A possible explanation for this striking dissociation in this patient is that altered neuronal recovery patterns following therapeutic hypothermia may lead to a disproportionate preservation of anterior forebrain cortico-thalamic circuits even in the setting of severe hypoxic injury to other brain areas. Compared to recent reports of other severely brain-injured subjects with such dissociation of clinically observable (overt) and covert behaviors, we propose that this case represents a potentially generalizable mechanism producing an isolation syndrome of blindness, motor paralysis, and retained cognition as a sequela of cardiac arrest and therapeutic hypothermia. Our findings further support that highly-preserved anterior cortico-thalamic integrity is associated with the presence of conscious awareness independent from the degree of injury to other brain areas.

## Introduction

Over the last 10 years, several studies reported that some severely brain-injured patients who appear to be in a vegetative or minimally conscious state (MCS) at the bedside, nevertheless show evidence for preservation of higher cognitive capabilities detected by functional neuroimaging or electrophysiological methods (Owen, 2006; Monti et al., 2010; Goldfine et al., 2011). This increasingly well-described phenomenon has been recently named as cognitive motor dissociation (CMD) (Schiff, 2015). Two independent studies (Monti et al., 2010; Forgacs et al., 2014) showed that ~10% of severely brain-injured patients may exhibit such dissociation between beside and functional neuroimaging findings, however, only a few studies addressed the important question of possible mechanisms underlying this syndrome (Fernández-Espejo et al., 2015).

Here we identify a novel isolation syndrome which may provide a generalizable mechanism for one type of CMD. This syndrome is heralded by the remarkable separation between high-degree preservation of anterior regions of the cortico-thalamic system and apparent severe neuronal loss in the sensorimotor regions. The clinical manifestations of the syndrome include blindness and severe motoric disabilities that preclude reliable responses to external stimuli or tracking of visual cues. In combination, these features lead to a high probability of a bedside diagnosis of vegetative state (VS) or MCS with retained possibility of preserved higher-integrative cognitive functions. Multi-modal neuroimaging and neurophysiological data below provide evidence for the mechanisms underlying this syndrome. We propose, that more generally CMD reflects the preservation of the anterior regions of the cortico-thalamic system and it is a possible sequela of cardiac arrest and altered neuronal recovery patterns following therapeutic hypothermia (TH).

## Background

### Clinical history

A 17-year-old woman, previously healthy with the exception of a single episode of syncope, collapsed while playing sports. She was found pulseless and cardio-pulmonary resuscitation was initiated immediately. Emergency medical services identified ventricular fibrillation, and she received multiple shocks. Total time to return of spontaneous circulation (ROSC) was prolonged at ~45 min. Equal and reactive pupils bilaterally were noted immediately after ROSC. She underwent standard therapeutic hypothermia protocol with 24 h of cooling with target temperature of 33°C followed by 24 h of rewarming.

A prolonged hospitalization and rehabilitation course followed with slow improvement of her neurological functions. She remained sedated and comatose for the first 2 weeks after the injury with spontaneous eye opening noted immediately with lifting of sedation. Multiple brief episodes of non-sustained ventricular tachycardia led to placement of an automatic implantable cardiac defibrillator (AICD) 1 month after the cardiac arrest, but comprehensive workup, including cardiac MRI, did not reveal a definite etiology of the cardiac arrest. Intermittent reports of the ability to move the right toes to command ~2 months post injury supported a diagnosis MCS. During a time period ~5 months after the cardiac arrest, she was inconsistently able to answer yes/no questions via eye opening. However, she soon after developed frequent episodes of autonomic instability including agitation and worsening severe spasticity, and lost this ability to communicate or follow commands using eye or toe movements. Subsequently, intermittent evidence of moaning, laughing, or smiling remained present with a stable neurological baseline.

Brain MRI at 4 days after the cardiac arrest revealed faint T2 hyperintensities in the occipital cortex and basal ganglia consistent with hypoxic-ischemic injury. Head CTs 4 and 6 months following the cardiac arrest showed overall volume loss, hypodensities in the medial and posterior aspects of the occipital lobes, bilateral basal ganglia, and lateral aspects of the bilateral putamina consistent with evolving encephalomalacia, but retained gray-white matter differentiation in other cortical areas.

The patient was studied 2 years 9 months after the cardiac arrest as part of an ongoing research protocol for disorders of consciousness.

### Ethics

This study was carried out in accordance with the recommendations and approval of the Institutional Review Board of The Rockefeller University, which abides by the Declaration of Helsinki. Informed consent was obtained from the legally authorized representative of the patient.

### Research evaluations and results

#### Behavioral examinations

Behavioral diagnosis was determined using Coma Recovery Scale-Revised (CRS-R) (Giacino et al., 2004), a quantitative behavioral assessment of level of consciousness in patients with severe brain injury with well-validated psychometric properties. The patient did not fixate on, or track visual stimuli, did not exhibit goal-directed movements, and did not have evidence of a communication channel. However, she intermittently appropriately laughed or vocalized sounds at the punch-line of jokes or funny stories. Therefore, her behavioral diagnosis of MCS was established based on the Assessment of Contingent Behavioral Supplement of the CRS-R.

#### [^18^F]-FDG-PET study

**Figure 1:**
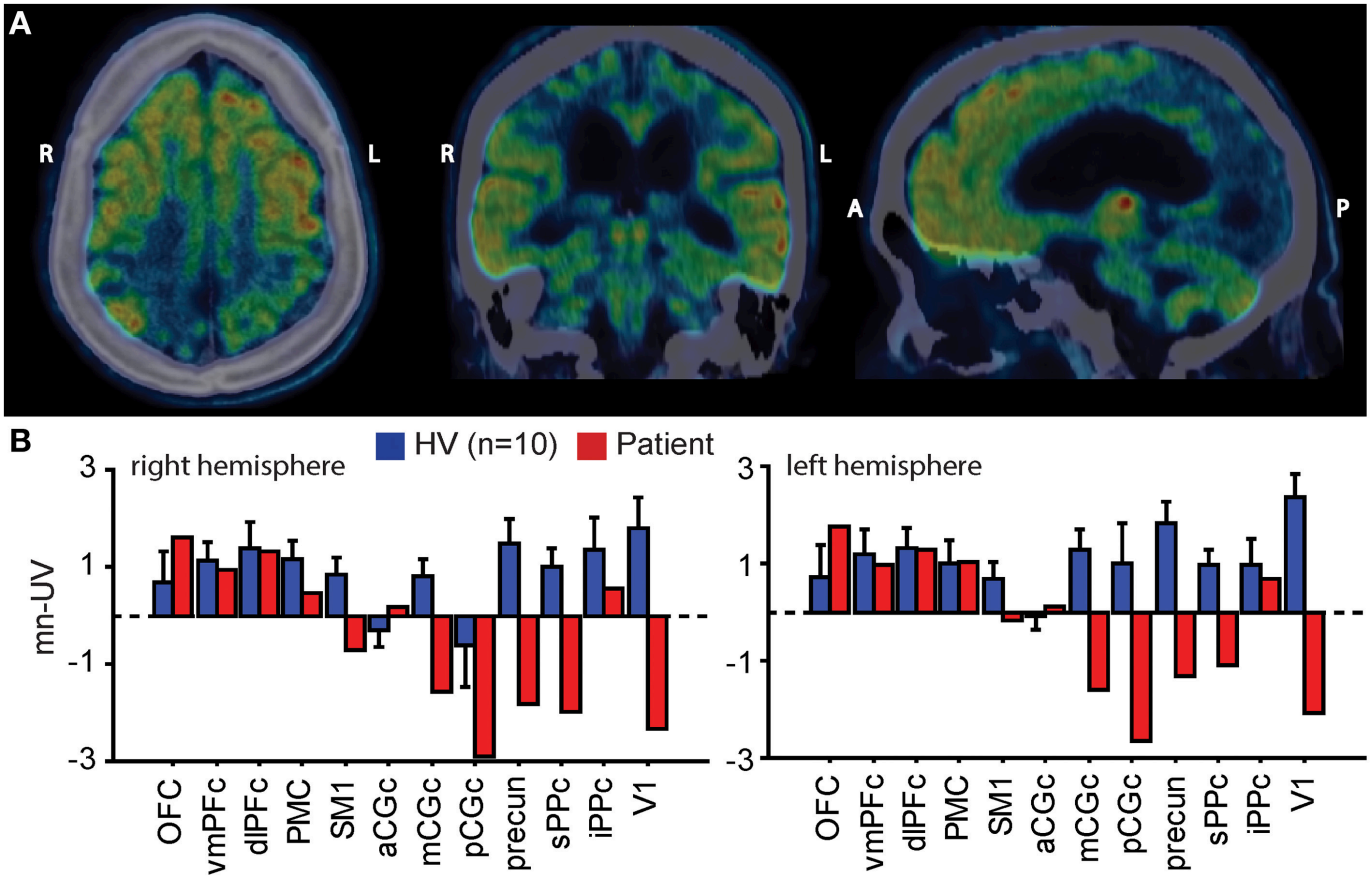
**(A)** Resting brain metabolism as measured by [^18^F]-FDG-PET superimposed on the patient's CT images in representative horizontal, coronal, and sagittal planes. Preservation of the anterior forebrain and bilateral thalami (yellow and red colors) and striatum (not visible on this image) with loss of posterior brain areas is evident. **(B)** Bar graphs show standard uptake values in selected cortical regions of interest scaled to the global mean (mean normalized uptake values; mn-UV) in 10 healthy volunteers (HV; blue columns, ±SD) and in the patient (red columns) in the right and left hemispheres. Mn-UV in the patient are normal in the anterior forebrain, including the prefrontal cortices and the left premotor area, however significantly decreased over the posterior cortical areas, including primary sensorimotor areas, bilateral parietal, and occipital cortices (primary visual area). R, right; L, left; A, anterior; P, posterior; OFC, orbitofrontal; vmPFC, dlPFC, ventromedial and dorsolateral prefrontal cortices; PMC, premotor cortex; SM1, primary sensorimotor cortex; aCGc, mCGc, pCGc, anterior, medial and posterior cingulate cortices; sPPC, iPPC, superior and inferior posterior parietal cortices; precun, precuneus; V1, primary visual cortex.

We measured resting brain metabolism using ^18^F-labeled fluoro-deoxy-glucose positron emission tomography [^18^F]-FDG-PET. Mean normalized uptake values (mn-UV) were computed in selected pre-defined cortical regions of interest (ROIs) (Tzourio-Mazoyer et al., 2002). We compared mn-UVs from the same ROIs derived from 10 healthy volunteers using the same methods (Fridman et al., 2014). [^18^F]-FDG-PET showed preserved brain metabolism throughout the prefrontal cortices and left premotor cortices. Bilateral primary sensorimotor cortices along with widespread areas in parietal and occipital lobes showed marked reduction of metabolic signal (Figure 1).

#### Resting EEG evaluations

**Figure 2:**
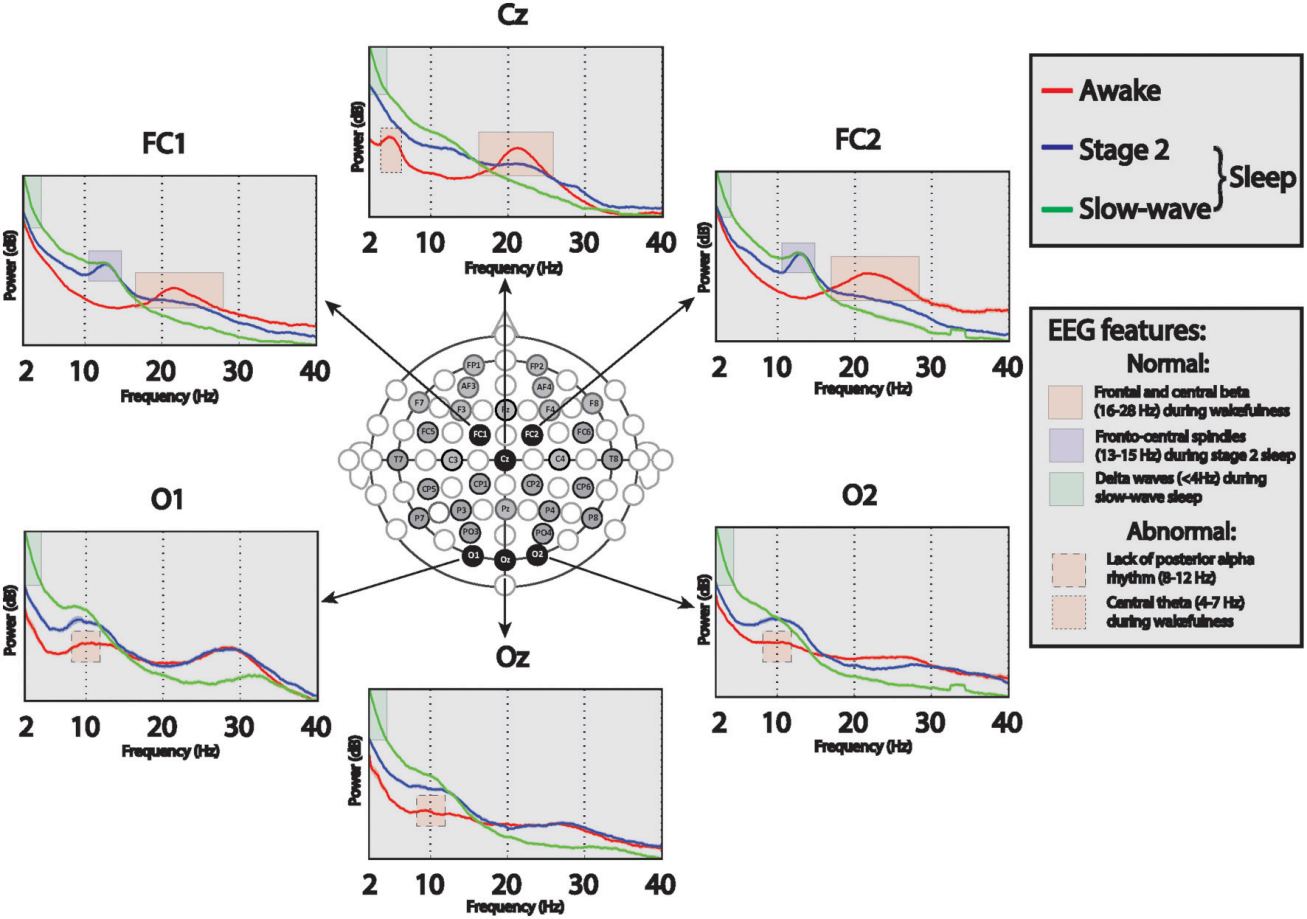
**Absolute EEG power frequency spectra in wakefulness (red lines), stage 2 sleep (blue lines), and slow-wave sleep (green lines) over select fronto-central and occipital channels**. During wakefulness the EEG demonstrates normal fronto-central beta (16–28 Hz; at Cz, FC1, FC2) activity, abnormally present central theta (4–7 Hz; at Cz) activity, and abnormal lack of posterior alpha (9–12 Hz; at Oz, O1, O2) activity, suggesting normal fronto-central and abnormal posterior brain functions. During stage 2 and slow wave sleep, sleep spindles (13–15 Hz; FC1, FC2) are seen maximally over fronto-central channels, and during slow-wave sleep increased delta (< 4 Hz, all electrodes) is seen.

Forty hours of continuous electroencephalogram (EEG) was recorded during the 2-day long research admission using a standard video-EEG system (Natus-XLTEK, San Carlos, CA). Sampling rate was 512 Hz, band pass filter settings were set between 1 and 70 Hz and notch filter was turned off. The EEG was recorded using 37 electrodes [Nihon Kohden (Japan) silver-collodion disc electrodes, 1.5 mm] placed by certified technicians according to an enhanced International 10–20 System. All EEG recordings were reviewed using a bipolar augmented double-banana montage; wakeful and sleep EEG features were assessed according to standard clinical neurophysiological conventions (Schomer and Lopes da Silva, 2010). To assist in the interpretation of the EEG in the identified awake and sleep stages, we performed power spectral analysis. Between 45 and 64 artifact-free, 10-s long epochs were selected for spectral analysis during the following states identified on the EEG by visual inspection: resting wakeful state with eyes open or closed (of note, no EEG background reactivity was observed in relation to eye opening), stage 2 sleep, and slow wave sleep. Power spectral density was computed for each channel using Thomson's multi-taper method (Thomson, 1982), as implemented by the code mtspectrumc in the Chronux toolbox (Mitra, 2008). Five tapers with frequency resolution of 2 Hz were used. Visual review and spectral analysis of the wakeful EEG showed characteristic frontal-central beta-range activity during wakefulness, and canonical features of stage 2 and slow wave sleep stages during sleep (Figure 2). However, the EEG also demonstrated an abnormal central theta feature and there was no posterior alpha rhythm during wakefulness (Figure 2).

#### Functional EEG evaluations

The presence of “covert” command following was assessed using previously published quantitative EEG methods (Goldfine et al., 2011) with minor modifications. Briefly, eight trials of pre-recorded commands stating “Keep opening and closing your right hand” (“move” condition) alternating with “Stop opening and closing your right hand” (“stop” condition) were presented to the patient via headphones during three recording sessions (“runs”) on two separate days of EEG monitoring. Before each run, the task was repeatedly explained to the patient and she was instructed to attempt a movement even if she was not able to perform an actual movement. Simultaneous video and lower arm surface electromyogram (EMG) was recorded to detect possible arm movements. There was no evidence of actual right hand movement on video or EMG recordings during any of the runs.

**Figure 3:**
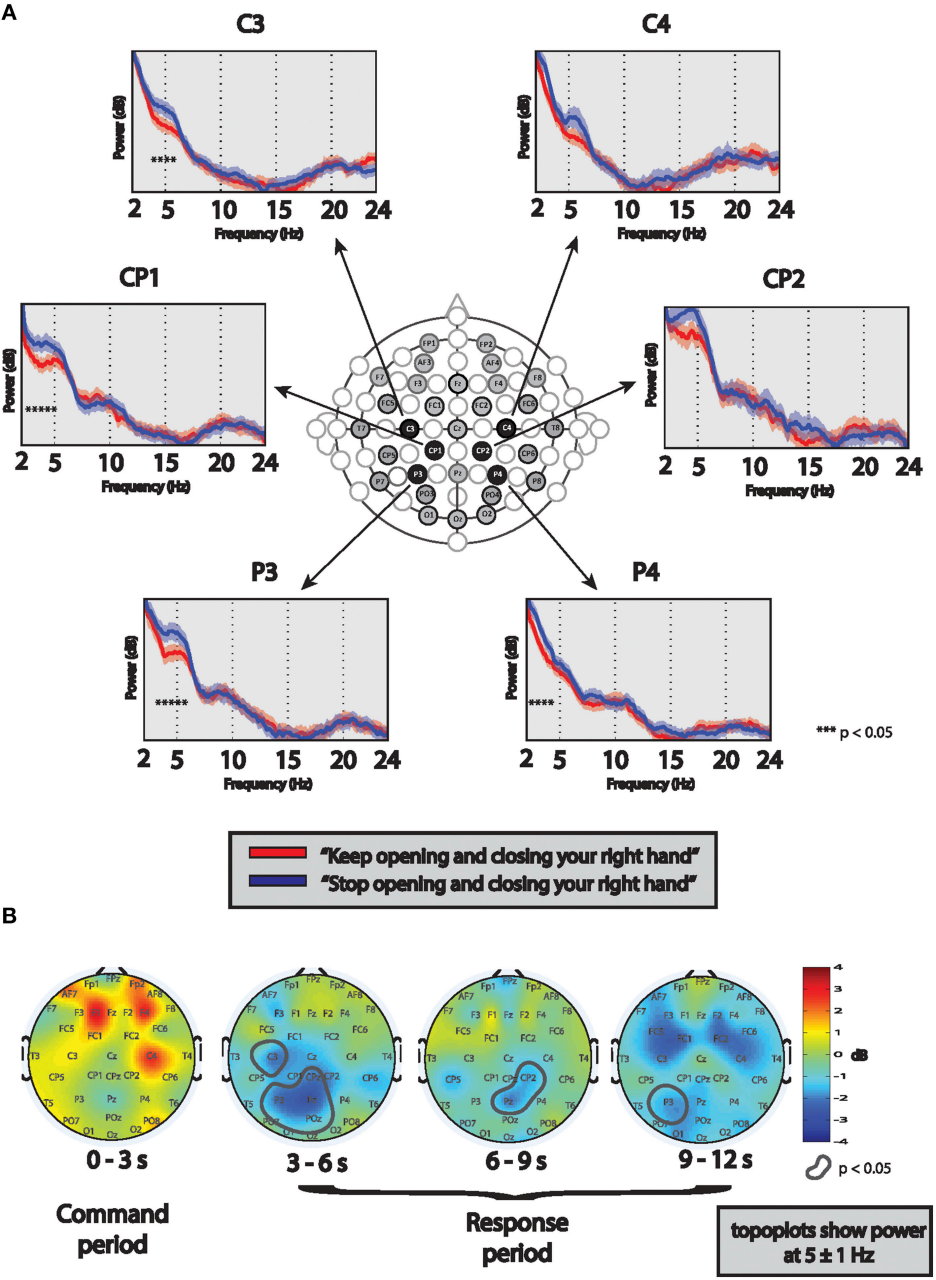
**(A)** Determination of command following using EEG: Spectra are based on 9 s-long EEG segments from each “move” (red lines) and “stop” (blue lines) trials after combining two runs (total of 16 commands in each condition). Significant differences in power spectral frequency content between conditions in each channel were determined using a *z*-test (*p* < 0.05; marked with stars). Significant differences are evident over left centro-parietal regions. **(B)** Determination of time course of signal differences: Topoplots show power differences between “move” and “stop” conditions at 5 ± 1 Hz frequency range in all channels in four 3-s long EEG segments. Red colors represent more power during trials of “move” condition, compared to “stop,” blue colors the opposite. Circled areas shows significant difference between the conditions at *p* < 0.05. During the first 3-s time segment (“command period”), some increased frontal power during the “move” condition is seen, however it does not reach significance. Significant differences between the conditions (with more power during “stop” conditions) are prominent throughout the “response period” in the three 3-s long time segments, however with slightly varying intensity and localization.

To determine presence of covert command following, 9-s long EEG segments starting 1 s after the end of each command were analyzed. After artifact removal, power spectral analysis was performed for each channel using the same methods as described above. For each run, significant differences in frequency content of the EEG between the task and rest conditions were determined using a *z*-test (Bokil et al., 2007) with a cutoff of *p* < 0.05 by jackknife method. The difference between “move” and “stop” conditions was deemed to be positive, if (1) at least two runs had significant results in the same channel and frequency, (2) after combining the runs with significant results identified at step 1, *p*-values remained significant after applying False Discovery Rate (FDR). In this patient, the above criteria met using runs #2 and #3; therefore the combination of these runs (a total of 16 trials in each condition) is presented here. Statistically significant differences in EEG spectral power between “move” and “stop” conditions localized over the left centro-parietal areas (Figure 3A), a similar pattern expected in healthy control subjects (contralateral electrodes over sensorimotor regions). However, in contrast to healthy controls in whom alpha suppression is typical (Pfurtscheller et al., 2006), suppression of the theta frequency band was observed in this patient, a feature which is also present in the patient's resting wakeful EEG spectra. Additionally, further analysis revealed that the signal appeared after the command ended and lasted throughout the response period with slightly varying intensity and localization (Figure 3B).

## Discussion

We describe a novel isolation syndrome in a young patient who sustained a cardiac arrest followed by prolonged resuscitation and standard TH protocol (Bernard et al., 2002). This syndrome is characterized by behavior consistent with low level MCS in this patient, yet widely preserved anterior forebrain neuronal metabolic activity demonstrated by FDG-PET, and preserved thalamo-cortical connections demonstrated by well-organized fronto-central resting wakeful and sleep EEG features. Identification of covert high-level cognitive functions is supported by the evidence of active modulation of brain activity in response to verbal commands. Bedside determination of awareness in patients with this syndrome is severely limited due to extensive loss of neurons across primary sensorimotor and visual cortices that may produce a total loss of motor control and blindness.

Two fundamental physiological mechanisms likely account for these findings: the selective vulnerability of defined neuronal populations to hypoxic-ischemic insults and the growing evidence that the anterior forebrain mesocircuit (Schiff, 2010) has an important role in maintaining normal consciousness.

While outcomes after cardiac arrest may generally reflect the consequences of global cerebral anoxia producing relatively uniform cellular death across brain regions, neurons with higher baseline metabolic activity, such as the primary motor and sensory cortices, bilateral basal ganglia and hippocampi are known to be more vulnerable to hypoxic conditions (Greer, 2006). At present, it is not known how variable exposure time to hypoxia leads to graded injury of vulnerable neuronal populations, and more importantly, how these injury patterns are potentially altered by TH. However, the sharp distinctions seen in our patient between preservation of anterior forebrain structures vs. the structural loss of primary sensorimotor and visual cortices and local metabolic suppression in surrounding regions, suggests that a threshold phenomenon of cellular rescue may arise with TH. These considerations are also supported by the recent evolution of clinical practice in neurological prognostication after cardiac arrest. Advancements in emergency and critical care lead to overturn of traditionally well-vetted clinical predictors for poor outcome after cardiac arrest (Levy et al., 1985; Booth et al., 2004; Wijdicks et al., 2006). Importantly, previously unexpected outcomes have only consistently surfaced after the introduction of targeted temperature management protocols (hypo- or normothermia) as standard of care in most clinical centers (Al Thenayan et al., 2008; Rossetti et al., 2010; Grossestreuer et al., 2013; Gold et al., 2014).

The preserved functional integrity of the anterior forebrain provides a specific mechanism and substrate for retained consciousness in this subject. Recovery from disorders of consciousness has been shown to correlate with restoration of central thalamic output to frontal cortex and striatum leading to modulation of widespread excitation across the anterior forebrain mesocircuit (Schiff, 2010; Fridman et al., 2014). Importantly, presence of sleep spindles and slow wave sleep in this patient further demonstrates the functional integrity of the anterior forebrain mesocircuit (Forgacs et al., 2014). Large population studies further support common physiological characteristics in patients with such cognition-motor dissociation that emphasize the crucial role of intact thalamo-cortical connections in patients with evidence of conscious awareness that is not recognized on bedside examination (Forgacs et al., 2014; Stender et al., 2014; Lutkenhoff et al., 2015). We also note, however, that the posterior medial complex of the parietal lobe has been severely injured bilaterally. This region is a critical component of the “default” mode network and the loss of these neurons may correlate with impaired higher level self-consciousness (Fingelkurts et al., 2012).

Our index case draws attention to a potentially very alarming generalizability of this clinical outcome after cardiac arrest secondary to the graded vulnerability of neuronal populations supporting visual functions and purposeful motor control. The findings warrant the careful assessment of all survivors of cardiac arrest treated with TH to rule out the preservation of higher cognitive functions. Our observations are further consistent with the inference that patients at risk of harboring significant cognitive reserve invariably show overwhelming injury to the motor system and highly-preserved brain structures across the rest of the corticothalamic system (Forgacs et al., 2014). Neurophysiological evaluation of patients with limited bedside behavior should be employed to identify possible preserved cognition even in the chronic stages of hypoxic/anoxic brain injury. In patients with cardiac pacemakers limiting functional MRI imaging options, EEG-based assessments and FDG-PET are the potential mainstay of evaluations.

## Concluding remarks

In summary, an isolation syndrome as described in this patient characterized by blindness with lack of executive motor functions and retained cognition is a possible sequela of cardiac arrest and TH. Our findings however, also highlight possible generalizable mechanisms of CMD. Previous studies involving larger cohort of severely brain-injured patients suggested preservation of widespread cortical metabolism (Stender et al., 2014) and global thalamo-cortical functions as reflected in sleep-wake EEG organization (Forgacs et al., 2014) to be used as a potential screening tool for the possibility of preserved cognition in these patients (Forgacs et al., 2015). Our findings are in agreement with previous studies, but distinctively emphasize that highly-preserved anterior forebrain integrity is required and sufficient to support conscious awareness even in the setting of severe injuries in other brain areas.

## Author contributions

PF, EF, AG, and NS contributed to the design of the work, the acquisition of the data as well as its analysis and interpretation, drafted, and revised the manuscript, approved the manuscript for publication, and agree to be accountable for all aspects of this work.

### Conflict of interest statement

The authors declare that the research was conducted in the absence of any commercial or financial relationships that could be construed as a potential conflict of interest.
